# Health diplomacy the adaptation of global health interventions to local needs in sub-Saharan Africa and Thailand: Evaluating findings from Project Accept (HPTN 043)

**DOI:** 10.1186/1471-2458-12-459

**Published:** 2012-06-20

**Authors:** Sebastian Kevany, Gertrude Khumalo-Sakutukwa, Oliver Murima, Alfred Chingono, Precious Modiba, Glenda Gray, Heidi Van Rooyen, Khalifa Mrumbi, Jessie Mbwambo, Surinda Kawichai, Suwat Chariyalertsak, Chonlisa Chariyalertsak, Elizabeth Paradza, Marta Mulawa, Kathryn Curran, Katherine Fritz, Stephen F Morin

**Affiliations:** 1 Institute for Health Policy Studies, University of California, San Francisco, 50 Beale Street, Suite 1300, San Francisco, CA, 94105, USA; 2 Center for AIDS Prevention Studies, University of California, San Francisco, USA; 3 University of Zimbabwe, Harare, Zimbabwe; 4 Perinatal Health Research Unit, Soweto, South Africa; 5 Human Sciences Research Council, Durban, South Africa; 6 Muhimbili University of Health and Allied Sciences, Dar es Salaam, Tanzania; 7 Chiang Mai University, Chiang Mai, Thailand; 8 Medical University of South Carolina, Charleston, USA; 9 Department of Epidemiology, University of Washington, Seattle, WA, USA; 10 International Center for Research on Women, Washington, DC

**Keywords:** Adaptations, Voluntary counseling and testing, Global health diplomacy, HIV, Sub-Saharan Africa

## Abstract

**Background:**

Study-based global health interventions, especially those that are conducted on an international or multi-site basis, frequently require site-specific adaptations in order to (1) respond to socio-cultural differences in risk determinants, (2) to make interventions more relevant to target population needs, and (3) in recognition of ‘global health diplomacy' issues. We report on the adaptations development, approval and implementation process from the Project Accept voluntary counseling and testing, community mobilization and post-test support services intervention.

**Methods:**

We reviewed all relevant documentation collected during the study intervention period (e.g. monthly progress reports; bi-annual steering committee presentations) and conducted a series of semi-structured interviews with project directors and between 12 and 23 field staff at each study site in South Africa, Zimbabwe, Thailand and Tanzania during 2009. Respondents were asked to describe (1) the adaptations development and approval process and (2) the most successful site-specific adaptations from the perspective of facilitating intervention implementation.

**Results:**

Across sites, proposed adaptations were identified by field staff and submitted to project directors for review on a formally planned basis. The cross-site intervention sub-committee then ensured fidelity to the study protocol before approval. Successfully-implemented adaptations included: intervention delivery adaptations (e.g. development of tailored counseling messages for immigrant labour groups in South Africa) political, environmental and infrastructural adaptations (e.g. use of local community centers as VCT venues in Zimbabwe); religious adaptations (e.g. dividing clients by gender in Muslim areas of Tanzania); economic adaptations (e.g. co-provision of income generating skills classes in Zimbabwe); epidemiological adaptations (e.g. provision of ‘youth-friendly’ services in South Africa, Zimbabwe and Tanzania), and social adaptations (e.g. modification of terminology to local dialects in Thailand: and adjustment of service delivery schedules to suit seasonal and daily work schedules across sites).

**Conclusions:**

Adaptation selection, development and approval during multi-site global health research studies should be a planned process that maintains fidelity to the study protocol. The successful implementation of appropriate site-specific adaptations may have important implications for intervention implementation, from both a service uptake and a global health diplomacy perspective.

## Background

### The HIV epidemic

From a public health perspective, there is no more compelling crisis in the world today than the HIV epidemic in sub-Saharan Africa. Since the epidemic began, more than 60 million people have been infected with HIV [1], and in 2009, AIDS killed 1.4 million people in Africa alone. In addition to the death and disease burden, the epidemic has had an enormous impact on economies, life expectancies, and society. Globally, about one-third of those currently living with HIV are aged 15–24, and young adults account for 40% of all new infections [1]. From the perspective of national AIDS control planners and policymakers in these countries, evidence-based strategies that have maximum epidemic impact on target regions and populations are critically important. For this to occur, both planners and implementers need interventions that are both sustainable, and adaptable, to the local epidemiological, cultural, economic and infrastructural context [2,3]. Randomized controlled trials that test such interventions should be correspondingly flexible and sensitive to local needs throughout their development and implementation.

### Adapting global health interventions

Adaptations to health programs are defined as the degree to which they are changed or modified by a user in the process of their adoption and implementation [4]. HIV-related interventions that have been proven for efficacy frequently need to be adapted to different environments and settings when implemented as public health programs [5,6]. Similarly, HIV-related trials, especially those that are conducted in multiple countries or at multiple sites within a country, frequently require site-specific adaptations to the intervention in order (1) to respond to socio-cultural differences [7] (2), to make interventions more relevant and sensitive to target populations (including different risk determinants and risk behaviors) [2,3,8], (3) to improve intervention utilization and effectiveness [9], and (4) to ensure that interventions meet minimum diplomatic [10] and foreign policy [11] standards. For the latter, an inability to align broader policy goals with global health initiatives, has the potential to create ‘a tense and confusing duality’ (CSIS, 2010). In all cases, adaptation development and implementation should be a planned process that maintains fidelity to core elements of the study protocol [12].

### Project Accept

The Project Accept intervention has been described elsewhere [9]. Briefly, 34 communities in sub-Saharan Africa (at study sites in Soweto and Vulindlela in South Africa, Kisarawe in Tanzania, and Mutoko in Zimbabwe) and 14 communities in Thailand were randomized to receive either a community-based voluntary counseling and testing (CBVCT) intervention in addition to standard clinic-based VCT (SVCT) services, or SVCT services alone. The CBVCT intervention had three major components designed to change community norms and reduce risk of HIV infection among all community members, irrespective of whether or not they participated directly in the intervention. These were (1) to make counseling and testing more available in community settings through mobile voluntary counseling and testing (MVCT), (2) to engage the community through community mobilization (CM), and (3) to provide post-test support services (PTSS), including psychosocial support groups, coping effectiveness and stigma reduction training. The intervention was of 42 months duration, with start and end-dates varying by site.

 Although the intervention in each of the countries and study sites was derived from the same theoretical model [13,14], contained the same core strategies, and conformed to common standard operating procedures, the implementation of intervention components was designed to be responsive and adaptable to each local context. To this end, all stages of intervention implementation were determined through intensive collaboration between host-country investigators and institutions, study communities, field staff, and their international partner institutions (listed at end of paper). In particular, the multisite study steering committee worked with representatives of national AIDS programs in host countries throughout the development of the intervention, on both a deliberative and an *ad-hoc* basis, both (1) to keep national planners informed on project activities and (2) to ensure that project activities were implemented in a manner that would be both appropriate and sustainable in resource-limited settings.

An intervention subcommittee was tasked with overseeing all aspects of the intervention and included members from each of the five project sites. The duties of the subcommittee were (1) to monitor intervention implementation to ensure that all sites worked to common standards, (2) develop and conduct regular quality assurance activities, and (3) propose, discuss and determine acceptability of site-specific adaptations to the study protocol. In addition, the intervention subcommittee established an intervention working group at each site including site principal investigators and project directors. These groups worked via conference calls and site visits throughout the intervention. This participatory process facilitated the resolution of both site-specific and cross‒site issues– and helped in developing, determining and applying protocol adaptations.

The CBVCT intervention was remarkably successful in terms of service utilization. After 42 months of the intervention, utilization of the CBVCT model was more than 10 times higher than utilization of SVCT in control communities , acriss sites [9]. This divergence was primarily attributable to the mobile nature of the intervention, combined with extensive CM efforts to promote utilization in intervention communities. In addition to these explanations, the ongoing adaptability of the CBVCT intervention to local conditions may have helped to generate and maintain these utilization levels.

### Adaptations to the Project Accept intervention

Each intervention component was designed with varying allowances for adaptations to local conditions throughout the intervention. For MVCT, sites were free to determine the most appropriate system of service delivery(e.g. tents, caravans, community buildings, or project vehicles) as well as field hours operating schedules. For CM, study staff were asked: ‘Is our innovation sensitive to, and compatible with, the community's existing values and beliefs, local culture, indigenous customs, past experiences, and the needs of potential adopters?’ In this way, CM staff were responsible for discovering how the intervention could be made more compatible with communities' existing values through (1) suggesting and developing new systems for working with local social networks and (2) the ongoing refinement of information, education and communication (IEC) messages. For PTSS, coordinators components were encouraged to work with community representatives to identify local post-test service needs as well as collaborating with related existing services (if any). For certain components of PTSS which had heretofore only been proved for effectiveness in industrialized countries, staff were encouraged to monitor their acceptability to local populations. In this paper, we report on the adaptations development and approval process at each site and the major adaptations developed and implemented at each site, presented according to (1) intervention component and (2) broader adaptations themes and types. For all components, while scope for possible adaptations was provided in advance of intervention implementation, the adaptation process itself was more precisely developed and ‘fine-tuned’, in response to specific (and often unforeseeable) issues, subsequent to intervention implementation.

### Ethical review

The study procedures were approved by the following ethical review committees: The Johns Hopkins University Committee on Human Research (Thailand); Chiang Mai University Research Institute for Health Sciences (Thailand); the Ministry of Public Health (Thailand); The Medical University of South Carolina Institutional Review Board (IRB) for Human Research (Tanzania); Muhimbili University of Health and Allied Sciences IRB (Tanzania); The National Institute of Medical Research IRB (Tanzania); The University of California, San Francisco Committee on Human Research (Zimbabwe); and The Medical Research Council of Zimbabwe (Zimbabwe); and the University of the Witwatersrand Health Sciences Research Ethics Committee (South Africa). Project Accept also had an independent Data Safety and Monitoring Board which biannually reviewed project benchmarks, outcomes, and adverse events. These ethical review committees remained active throughout the intervention, reviewing and advising the study steering committee on specific proposed intervention adaptations as part of the approval process. In turn, the project-specific ethics committee was composed of investigators from each site, and advised on when an adaptation would require a protocol modification to be approved by these review boards.

## Methods

### Community consultations and adaptation implementation

Monitoring community members' attitudes toward study activities and promoting community involvement were key elements of intervention development and helped study staff to troubleshoot implementation problems and implement adaptations with the support of intervention communities. Throughout the duration of the study, structured community involvement helped (1) to ensure ongoing two-way communication between study teams and study communities and (2) to respond to the ethical and practical issues raised by trial participants or their representatives. Communication with relevant district, regional and national leadership, including local government representatives, was also maintained throughout the intervention as required. A number of ways of maintaining community involvement in the adaptations process were developed, as described below.

### Community working groups

Community Working Groups (CWGs) represented community members' interests in each intervention community. CWGs consisted of approximately 30 representatives from a cross-section of local ethnic, tribal and community groups who were also leading members of local social networks, including faith-based organizations (FBOs), sporting clubs, community health services, and local government. CWG members were encouraged to keep in close contact with their communities, and frequently met in the absence of the research team to solicit feedback on project activities that the community might be reluctant to articulate in a larger forum. CWGs also helped to develop and tailor information, education and communication (IEC) materials relevant to the various social networks within the community and were supported through continuing training and education throughout the intervention. CWG members were not project staff, but were paid a sitting allowance at some sites for attendance in meetings. Representatives from each CWG also served on a Study Advisory Committee (SAC), designed (1) to allow primary stakeholders to exercise leadership at a higher level of study coordination and (2) to ensure community involvement both within and across communities.

### Community-based outreach volunteers

As part of the CM team, community-based outreach volunteers (CBOVs) were responsible for liaising with local organizations such as peer groups, FBOs and social clubs on behalf of Project Accept. CBOVs were community members who became early adopters of VCT or PTSS and, where possible, held strategic positions within local social networks. As community members, CBOVs were uniquely placed to monitor and report on the ongoing acceptability of the intervention to participating communities.

### Community engagement

Study staff were consistently encouraged to develop new and creative ways to keep participating communities informed about, and engaged in, intervention development, acceptability and adaptation. These included regular, interactive community meetings; the creation and use of informal communication channels (e.g. impromptu group or individual discussions with community members); developing relationships with key informants outside CWG structures (e.g. local business owners); and facilitating linkages between CWGs other community organizations.

### The adaptations process

While provision for adaptations was made in the original study protocol, as described above, the adaptations process was primarily developed during the intervention itself, on a site-by-site, ‘learning by doing’ basis, as appropriate to the wide range of issues and often unforeseen obstacles that had to be addressed throughout the intervention period. The adaptations process therefore evolved organically, throughout the intervention, on an *expost* rather than on an *ex-ante* basis. In this way, the use of community involvement and feedback mechanisms were essential to the adaptations process across sites. Key features of adaptations development and implementation were consistent across sites, and included (1) identification and discussion of site-specific utilization and uptake issues on bi-weekly conference calls with the intervention sub-committee throughout the intervention period in order to ensure prompt feedback on utilization challenges; (2) submission of a written proposal for adaptation to the intervention sub-committee (3); review of proposals, recommendations, and forwarding to the steering committee for further review; and (4) the steering committee making a final decision on whether to approve or reject the proposed adaptations. As described above, the CBVCT intervention also maintained an ethics sub-committee throughout the intervention period, responsible for the review and approval of any proposed adaptations to service delivery from a human subjects perspective, in order to determine if protocol modifications would need to be approved by the various review boards. Adaptations approved by the ethics and intervention sub-committees, and later by the study steering committee, were then introduced to project staff via periodical cross-site retrainings and intervention sub-committee site visits. Project coordinators were then responsible for pilot-testing the adaptation and, equally importantly monitoring performance of field staff as the adaptation was implemented.

### Zimbabwe

In Zimbabwe, adaptations were initially proposed by field staff during weekly debriefings following consultations with and feedback from CWGs, MVCT, CM and PTSS participants, as well as other stakeholders in the study communities, including ward councilors, village heads, local chiefs, and religious and political leaders. Proposed adaptations were then discussed at internal project management meetings with the principal investigator, and approved adaptations were discussed with intervention monitors and the steering committee before implementation. Approved adaptations were then communicated to the local chief community, who then instructed junior chiefs, village heads, and so on down the local hierarchy to assist project staff with their implementation.

### Thailand

In Thailand, field staff across components and CWGs were responsible for observing intervention acceptability and performance on a day-to-day basis throughout the intervention. Additional community-level consultations with teenagers, village health personnel, religious groups, households, and other local volunteer or charity groups were also conducted throughout the intervention. Project staff reported findings and suggestions for adaptations to the project director at weekly project meetings, which were in turn considered at monthly meetings of senior staff, including local principal investigators, the project director, and intervention component coordinators. Implementation of adaptations was rolled-out to the broader community after consultation with CWGs and other local stake holders, including community representatives from government, health care and commerce. CWG meetings were frequently divided into smaller working groups to discuss the implementation of proposed adaptations and, were attended by both senior and field staff.

### Tanzania

In Tanzania adaptation conceptualization and development took place at the field staff and CBOV level. Based on their day-to-day experience of field activities, meetings between field staff and CBOVs were held on a regular basis to exchange ideas on intervention implementation. Proposed adaptations were then referred to component coordinators, the local project director, the SAC, and the local principal investigator. After preliminary approval for adaptations from the study steering committee, further approval was sought from local religious leaders and community groups. Before implementation, all adaptations were publicized and explained in community meetings.

### Vulindlela

Project staff initiated changes to the intervention in Vulindlela. The site used an ongoing data management and fieldwork ‘feedback loop’ to monitor intervention implementation and adaptations, and weekly review and planning meetings between field staff and senior management were central to ensuring efficiency and effectiveness of adaptation delivery. Using monitoring and evaluation data, including utilization and quality assurance measures to track operational innovation effectiveness was also critical to the adaptations process. CBOVs also played an important role at the Vulindlela site, providing (1) a useful source of ideas for what innovations would or would not work in this cultural context, and (2) ensuring that adaptations obtained the necessary support from traditional, political and community structures.

### Soweto

In Soweto, adaptations to the intervention originated primarily with field staff. Cross-component events planning committees made up of MVCT, CM and PTSS representatives were established in each intervention community to develop adaptations before presentation to the project director. Adaptations project leaders were then identified by the project director and component coordinators and were responsible for providing organizational leadership in the implementation of adaptations. CBOVs were essential to the implementation of adaptations in Soweto by generating support from the primary stakeholders, including local NGOs, in the community.

## Results

### Cross-component adaptations

#### Intervention delivery adaptations

Adaptations to the structure and schedules of field teams were required across sites. In Zimbabwe and Vulindlela, the initial division and rotation of field teams across intervention communities by intervention component (MVCT, CM and PTSS) was found to be both inefficient in terms of staff transportation and confusing to participants. In Zimbabwe, separate CM, MVCT and PTSS teams were restructured into two combined teams, each of which included representatives from MVCT, CM and PTSS. Each team was allocated two intervention communities, and operated in each community for a two-week period each month. In Vulindlela, four combined teams were created and assigned to individual communities for the remainder of the intervention. Intervention delivery schedules were also adapted to local conditions. In Zimbabwe, project staff negotiated with mining employers to deliver the intervention at workplaces for clients that would not otherwise have had access to project services during working hours, and, similarly, with farming employers during harvest times. In Thailand, it was observed within the first 6 months of the intervention that utilization by the 18 to 32 year-old target population was higher on evenings and weekends, and intervention delivery was rescheduled for these times. In Vulindlela, a late afternoon and early evening intervention delivery schedule was introduced in response to participant feedback and based on a successful pilot. Weekend service delivery was also piloted, but abandoned after participants reported a preference for attending family activities in Vulindlela and as a result of low demand in Tanzania.

A number of other cross-component adaptations were also introduced throughout the intervention. In Vulindlela, a performance incentive system, including bonuses for staff meeting intervention delivery targets was introduced in response to high levels of staff turnover in the early stages of the intervention. The risk of coercion was mitigated by the exclusion of field supervisors from this incentive scheme. Also in Vulindlela, a reluctance by participants to evaluate project activities using written forms meant that these were replaced by feedback discussions with CBOVs. In Thailand and Zimbabwe, the frequent attendance of intoxicated villagers at intervention activities meant that the sites had to develop a system to assess eligibility for participation based on the amount of alcohol consumed. In Tanzania, owing to the long distances involved, project equipment was stored in secure village storage areas, including the houses of village leaders.

#### Religious adaptations

The support of FBOs can be critical to the acceptance of HIV interventions in resource-limited settings [15,16]. Sites were encouraged to respond to objections, suggestions or concerns from FBOs about project activities as they arose, and to adapt the intervention accordingly. Across sites, interaction with FBOs included project staff facilitating HIV/AIDS discussions during religious services, provision of MVCT on religious center grounds, and the establishment of FBO-based support groups. In Zimbabwe, project staff proactively engaged with religious groups with known histories of discouraging members from seeking medical services. In those cases where religious leaders declined all involvement with the intervention, their decision was respected by study staff. In Thailand, support from local monks was essential to community acceptance of the intervention in Buddhist communities, and staff were trained in the correct etiquette and protocol for working with senior religious figures. In addition, monks in intervention communities were invited to participate in training sessions on project goals and the impact of HIV on their communities. As a result of these efforts, Buddhist temples were made available for project activities, and a number of monks worked closely with CBOVs. In Zimbabwe and Thailand, the discussion of religiously-sensitive issues was discouraged during project activities held in, or in close proximity to, Catholic churches (e.g. on church grounds). In Soweto, special sessions for local Christian church leaders (*abafundisi*) were introduced to promote awareness of project activities: as a result, religious leaders both accessed MVCT and disclosed their HIV status during *bafundisi* forums. In Tanzania, intervention activities were divided by gender in Muslim areas, project activities were not provided in mosques, and CM messages were adapted to ensure sensitivity to local Muslim communities, including a suspension of services during Ramadan when requested.

#### Epidemiological adaptations

Sites were encouraged to adapt intervention delivery to respond to the local HIV epidemic, and in particular to target those high-risk populations with low service utilization. In Zimbabwe, Tanzania and Soweto, high levels of HIV prevalence amongst younger age groups [17] led to the introduction of ‘youth-friendly’ activities and curricula, including intervention presence at soccer tournaments, where information sessions were provided before and during matches; presentations on project activities in school guidance and counseling classes; inter-school quiz competitions on HIV and project activities; and the development of support groups for out-of-school youths. In Zimbabwe, the intervention targeted truck drivers and roadside vendors (via intervention delivery at halting sites); military personnel (via information sessions at local barracks); and couples (via promotions encouraging joint MVCT and PTSS attendance). In Thailand, where the HIV epidemic is concentrated in young, high-risk populations [1,18], CBOVs held workshops with teenage groups to assess which aspects of the intervention were most attractive to them. In Soweto, specific population groups were targeted for mobilization, including traditional healers, Zulu males living in hostels, and women's groups.

#### Social, political & cultural adaptations

Although efforts had been made to include site-specific social and cultural adaptations during protocol development, a range of further and related adaptations were introduced throughout the intervention through the identification of popular local practices by field staff. Across all sites, popular social gathering places (e.g. pubs and community centers) and their times of peak operation were identified over time and included as intervention venues. In Zimbabwe, Tanzania and Soweto, adaptations were made to health and project terminology based on feedback from participants and in keeping with local dialects. In Thailand, these included tribal rituals (e.g. sword dances and costume displays) performed during project events; field staff hired based on their knowledge of local languages and customs; the provision of karaoke facilities to participants during evening sessions; and special liaison staff hired and trained to work with refugee groups residing in intervention communities located on the Thailand-Burma border. In Tanzania, cultural norms meant that women were discouraged from utilizing project services. As a result, targeted campaigns at female gathering places (e.g. water boreholes) to encourage women to access project services were introduced. Also in Tanzania, the intervention was re-named as “Project Afiki” (the local Kiswahili term for “Accept”). In both Thailand and Tanzania, small gifts are frequently exchanged in social settings as a form of etiquette. Although the protocol prohibited the provision of material incentives to attend project activities, it was agreed that the provision of small, valueless presents (e.g. key rings) to participants would be permitted as a cultural concession. In Soweto, field staff observed that many participants were reluctant to access MVCT services alone and preferred to participate in groups. As a result, a ‘Bring a Friend' strategy was introduced.

### Mobile voluntary counseling and testing

#### Intervention delivery adaptations

Ongoing adaptations to MVCT delivery were required across sites, and counseling messages were updated to reflect both new scientific evidence and local needs throughout the intervention. Across sites, (1) culturally-tailored health services messages were introduced into counseling curricula, including information on local availability and referrals (except practices); (2) treatment referral messages were regularly updated to reflect the ongoing expansion of antiretroviral therapy, and (3) special counseling curricula for high-risk population groups were delivered in response to emerging evidence on their role as drivers of the epidemic. In Zimbabwe, clients requested, and recieved the addition of a bereavement-counseling message to assist in coping with HIV-related deaths.

MVCT adaptations according to ongoing changes in national health policies were also required across sites. In Tanzania, project staff coordinated MVCT delivery with the president's national testing campaign [19], which was rolled out for a three-month period midway through the intervention. In Soweto and Vulindlela, the minimum age of participation in MVCT was lowered from 18 to 16 years old, in keeping with the South African national HIV testing policy [20].

#### Environmental and infrastructural adaptations

Across sites, MVCT venues and equipment were adapted to local environment and infrastructure. In Zimbabwe, remote villages were frequently inaccessible due to road and weather conditions, and participants reported walking distances of up to 7 kilometers to access designated venues. In response, intervention teams introduced MVCT to a series of remote ‘satellite venues' identified in collaboration with CBOVs. In Thailand, the communal philosophy of ‘house, temple, school' was adopted for MVCT delivery. With the permission of local community leaders, Buddhist temples, Christian churches, other religious centers, private houses, and local health centers were used as MVCT venues, and river-boats and motorbikes were used to transport MVCT staff to more remote venues. Tables and chairs were provided by the communal temple (*wat*), and community members were active in helping to set up equipment. As a result of this community assistance, the site did not need to invest in caravans or tents throughout the intervention. In Tanzania, roadside service provision was introduced along major transportation routes to increase MVCT utilization. In Vulindlela, Soweto and Zimbabwe, and with the encouragement of teachers, MVCT was provided in local schools. CM staff later reported increased knowledge and awareness of project activities from students' parents. In Vulindlela, the original MVCT caravans were replaced with larger models in order to meet utilization demands at peak times, while in Zimbabwe and Tanzania, tents were used instead of caravans for less accessible sites.

### Post-test support services

#### Intervention delivery adaptations

Community perceptions of PTSS were ambivalent at the introduction of the intervention and PTSS participation was often mistakenly associated with HIV infection by community members. In these cases, community members assumed that only HIV-positive individuals would require PTSS. The resulting initial low uptake of PTSS drove a range of adaptations to eligibility and participation criteria to improve both recruitment and community perceptions of PTSS. Across sites, initial uptake of PTSS services was mainly by people who had not yet been tested for HIV by Project Accept. These individuals were designated as “guests” within the PTSS system and were granted limited access to services. This restriction was abandoned after ‘guest’ participants requested greater access to PTSS in order to *prepare* for VCT. As a result, the role of PTSS evolved into supporting and advising both VCT participants and those considering VCT. In Zimbabwe, where PTSS groups were initially separated between HIV positive and negative individuals, combined-status groups were introduced, and participants were invited both to contribute to curriculum development and form their own administrative structures.

The structure, content and scheduling of PTSS sessions were also adapted in response to participant demands. Across sites, individual psycho-social counseling sessions were initially only available by advance appointment. As the intervention progressed, and in response to client demand, this service was were provided before and after other PTSS activities, on an *ad-hoc* basis. In Thailand, (1) CET sessions were divided by age group to accommodate a wide age range of participants, each of which had different discussion preferences, and (2) support group provision was delivered in collaboration with local government health centers after staff discovered that these centers were delivering an identical service. Also in Thailand, due to the low incidence of HIV in intervention communities, CET sessions were adapted to provide information to HIV-negative persons on coping with the potential risk of HIV and helping others to cope with HIV. In Thailand and Tanzania, the planned 8-hour sessions for coping effectiveness (CET) and stigma reduction activities were found to be too long for participants, and were reduced to three 3 hour sessions over successive days. In Tanzania, more flexible scheduling of PTSS was introduced to respond to community demands.

Various other adaptations were made to PTSS service delivery throughout the course of the intervention. Across sites, PTSS information booths were set up adjacent to MVCT to facilitate recruitment of participants to PTSS. In Tanzania, sporting activities, card games and board games were introduced into PTSS sessions in order to provide a social forum for HIV discussions. In Zimbabwe, a trainer-of-trainers course was introduced for PTSS participants. In Soweto, PTSS advocates were trained in safe disclosure procedures, and special PTSS sessions designed for the education and training of traditional healers on HIV/AIDS and related issues were introduced. As a result, a number of traditional healers referred their patients to MVCT and PTSS. In Vulindlela, PTSS staff provided telephone reminders of session times to new participants.

#### Environmental and infrastructural adaptations

Across sites, PTSS venues were adapted to the local environment and infrastructure. In Zimbabwe, Vulindlela and Soweto, PTSS was redesigned as a mobile service in order to improve access. Venues included schools, community halls, and local business centers. In Vulindlela, participants indicated a preference for accessing PTSS in communities other than those in which they lived, in order to reduce possible stigma associated with PTSS participation. As a result, Project Accept offices, which were located outside intervention communities, were adopted as PTSS venues. Transport was provided to participants by project staff, and participants subsequently reported a more enabling and open learning environment. In Zimbabwe, participants reported difficulty accessing PTSS referral services due to transport costs. In response, project staff transported service providers, including nutritionists, lawyers, and nurses, to the intervention communities.

#### Economic adaptations

PTSS adaptations also responded to local economic conditions. In Zimbabwe and Tanzania, poverty, hunger and malnutrition were common issues in intervention communities and were frequently cited as reasons for non-attendance of longer sessions. The following adaptations were developed in response: (1) provision of tea, lunch and nutritious drinks (*mahewu*) to participants, (2) income generation and skills development classes provided before and after PTSS activities, (3) development of partnerships with local organizations and government officials to provide farming inputs, food aid, and legal services to participants, (4) support groups were provided with horticultural equipment and training in partnership with local groups, and (5) lay counselors were trained through the Zimbabwe Institute of Systemic Therapy (ZIST-CONNECT). In Thailand and Tanzania, income-generating equipment, including chicken coops and crop seeds, were provided to both unemployed and HIV-positive participants. These adaptations helped both to mitigate the economic effects of HIV-related stigma and to attract HIV-positive persons without other means of income to PTSS.

### Community mobilization

#### Intervention delivery adaptations

Across all sites, ongoing adaptations to CM messages were required to mitigate both participant fears of blood draws and intervention ‘staff stigma’, in which project staff were suspected of being infected with HIV. In Vulindlela, the use of pamphlets as an outreach strategy was found to be ineffectual due to poor literacy and was replaced by public mobilization talks held in central and public community areas. In Soweto, CM ‘road shows’, which involved vehicles and field staff touring through intervention communities, were developed and included a combination of door-to-door activities and participation by prominent local community members; CM ‘street dialogues’, which involved approaching and engaging community members in conversation about the intervention, combined with the distribution of promotional materials; and skills development workshops, including career counseling, were provided to community members as part of CM activities. In both South African sites, megaphones and loud hailers were used to deliver CM messages and announce MVCT and PTSS venues and activities after staff observed community members using PA systems to announce community events. In addition, youth-specific CM activities were introduced in schools, and project drivers were trained to become part-time CM staff.

#### Environmental, political and infrastructural adaptations

CM activities were continually responsive to the local environment. Across all sites, and in response to community feedback, CM activities were gradually relocated closer to MVCT and PTSS venues, throughout the intervention. In Zimbabwe, (1) CM activities were suspended during election periods to avoid being mistaken for political activism and ensure the safety of staff, participants and project equipment, and (2) CM staff provided transport and nursing staff wherever possible during disease outbreaks (e.g. cholera), which was beyond the initial scope of the protocol. In Soweto, CM campaigns specifically focused their efforts on Zulu hostels, which were initially opposed to male involvement in HIV testing activities, and, in both South African sites, CM staff promoted the intervention as a part of the ‘‘life skills’‘ curriculum in local schools. In Tanzania, CM activities in remote communities were concentrated in the dry season, and CM team size was adjusted across communities in inverse proportion to intervention demand.

#### Community-based outreach volunteers

Across sites, the role of community-based outreach volunteers (CBOVs) evolved in response to intervention needs. In Vulindlela, unanticipated demands of CM work on field staff resulted in the increased recruitment and utilization of CBOVs. CBOVs were given individual and group targets for PTSS and MVCT recruitment, and stipends, transport allowances, and other incentives were provided.As a result, CBOVs were renamed Community-Based Outreach Mobilizers in keeping with their enhanced CM role. Also in Vulindlela, a strong family culture led to the deliberate recruitment of CBOVs from large families, who then encouraged their social networks to attend MVCT and PTSS. In Zimbabwe, initial low levels of involvement by women prompted the training of increased numbers of female CBOVs. In Tanzania, CBOVs helped to advise on the choice of intervention venues, giving them an unanticipated role in the intervention delivery process.

## Discussion & conclusions

### Consistency & flexibility in adaptation development

Both the adaptations process and implementation of the adaptations themselves were critical to the acceptance, utilization and sustainability of the Project Accept intervention across study sites. The adaptations process, which had to balance sensitivity to the local context in order to improve utilization against consistency in the core elements of intervention delivery in accordance with the multi-site study protocol, also needed to ensure a minimum level of comparability across sites. In this way, site-specific adaptations processes, while procedurally variable, maintained a common set of features. These included (1) involvement of field staff in the generation of adaptations, (2) community acceptance measures, and (3) the role of the study steering committee, ethical review boards, and intervention sub-committees in their approval and implementation.

The adaptations developed across sites, while diverse, also maintained a common set of themes and approaches. In many cases, the same cross-component, or component-specific, adaptations would be developed independently across sites. In other cases, successful adaptations in one site would quickly be adopted by other sites. In these ways, a number of common themes in adaptation types, such as those identified above, were readily identifiable across sites.

### Measuring adaptation impact & estimating effects on uptake

 While our experiences, and associated anecdotal evidence, provide compelling support for the hypothesis that these adaptations influenced the impact of the CBVCT intervention through increased MVCT and PTSS utilization, it is, of course, not possible to establish a purely causal link between adaptation implementation and such outcomes. A range of potential confounding factors exist, including, but by no means limited to, the stage of intervention implementation, increased community acceptance over time, and broader environmental changes in knowledge of, and attitudes towards, testing for HIV in intervention communities. Nonetheless, is it notable that intervention uptake in intervention communities was over 10 times higher than in control communities, in which no adaptations were permitted, by the end of the intervention [9]. In addition, service utilization monitoring and evaluation data, when reviewed on a site-by-site basis, may be chronologically associated with the implementation of significant adaptations, when allowing for appropriate time lags. For example, at the Vulindlela site, service uptake for MVCT rose by over 100 per cent (mean uptake for the first two quarters of 2007 was 142 participants, as compared to 289 participants in the second two quarters, and increasing further to 369 participants in the first two quarters of 2008), directly after the service provision plan was adapted from rotating specialized MVCT, PTSS and CM teams to community-based, integrated, and multi-skilled teams. Given the temporal proximity of such improvements in utilization with adaptation implementation, it may be postulated that such adaptations may have had, across sites, a significant impact on service utilization.

### Determining when to challenge societal norms

One of the most challenging aspects of the CBVCT intervention was determining when to challenge societal norms in intervention delivery and adaptation. For example, as described above, gender issues were circumvented through the adapted delivery of female-specific interventions. On the other hand, religious issues were, in general, accommodated, rather than challenged, by adapting the intervention. Decisions around when societal norms should be challenged, through intervention adaptations are inherently subjective and environment-specific [14]. However, as a general principle, adapting to, rather than challenging, societal norms was found to be most appropriate in the CBVCT context.

### Diplomacy, global health, and foreign policy

The capacity to adapt global health interventions to local conditions also has considerable significance from the foreign policy perspective, in the context of the discipline now known and recognized as global health diplomacy [10]. Under this aegis, the inclusion of scope for adaptability in intervention design and delivery has the potential to make significant differences to local perceptions of international development activities, including the approval and acceptance of programs and interventions by recipient countries, communities and individuals, and, by extension, the profile and prestige of donor organizations and countries [21,22]. Over time,such interventions may need to become more sensitized to these broader roles and responsibilities, as currently reflected at the policy level by increased integration between departments of foreign policy and foreign assistance in donor countries - helping to maintain, or even increase, levels of development funding on a ‘smart power’ basis. Systems of more formallly considering the value and worth of global health interventions from a ‘foreign policy’ perspective may be developed, including such considerations as community acceptance and involvement, so that such they can be monitored and evaluated on this basis [23]. The associated capacity of interventions to adapt to local economic, cultural, religious and other environmental conditions in recipient countries may have the potential to make a substantial difference to those broader ‘collateral’diplomatic and, diplomatic and foreign policy outcomes of foreign assistance programs [24].

### Recommendations

A number of adaptation-related recommendations for related future interventions may be gleaned from the Project Accept experience. Collaboration with local actors, not just in service delivery, but at the highest levels of intervention design and planning, is, of course, a *sine qua non* of any enlightened intervention in the 21^st^ century. Perhaps most importantly, however, the necessity of building in provisions and scope for adaptations to the study in advance of intervention implementation, on an *ex ante* basis, should be borne in mind both by planners and scientists. This may be achieved, most directly, through the use of appropriate terminology in both the study protocol and standard operating procedures documents: in avoiding the use of dogmatic excessively or didactic language, interventions may be implicitly permitted appropriate levels of flexibility in intervention delivery, whilst maintaining fidelity to the original study protocol. Similarly, related and future studies should, wherever possible, make allowance for the monitoring and evaluation of changes in service utilization consequent to adaptation implementation- though as described above, this may be difficult to achieve in the presence of multiple competing factors effecting service uptake. Nonetheless, wherever possible, the effect of adaptation implementation on key study outcomes should be carefully reviewed, on a site-by-site basis, and with reference to appropriate lead times, in order to determine their effectiveness. Finally, as described above, the broader ‘collateral’ gains of such adaptations, such as improvements in international relations and community acceptance, should be carefully considered, wherever possible, through the development and application of appropriate metrics.

## Conclusions

Adaptations are particularly important in the design of HIV-related interventions. Given the oft-contentious nature of such interventions from a social, cultural and religious perspective [25], it is essential to ensure that, wherever possible and from both the service delivery and the global health diplomacy perspectives, these interventions are designed with the expectations and needs of recipient communities in mind. In the more specific case of VCT, for which utilization by most-at-risk populations is frequently a key issue [26], adaptability to local conditions may help to break down a both behavioral and environmental barriers to the testing process.

Ultimately, the need for intervention adaptation is based on challenges that could not have been foreseen during the design and launch of the intervention. Preparations will never be perfect, so time, money, capacity and flexibility to produce interventions has to be ‘built-in’ to the design of both trials, and interventions themselves, at all stages of the planning process. Without this inherent adaptability, program designers and implementers risk achieving sub-optimal utilization levels. As long as integrity to the key elements of the intervention protocol can be maintained, such adaptations can only enhance the value of the intervention in the eyes of policymakers, communities and individuals - in both donor and recipient countries.

## Competing interests

The authors declare that they have no competing interests.

## Pre-publication history

The pre-publication history for this paper can be accessed here:

http://www.biomedcentral.com/1471-2458/12/459/prepub
